# Fibrosis-4 index can predict improved renal function in acute heart failure with preserved ejection fraction

**DOI:** 10.1007/s10157-025-02669-w

**Published:** 2025-04-07

**Authors:** Koken Irie, Yosuke Watanabe, Manabu Uematsu, Hiroshi Yokomichi, Yuma Ichikawa, Takeo Horikoshi, Toru Yoshizaki, Juntaro Deyama, Kenji Kuroki, Tsuyoshi Kobayashi, Takamitsu Nakamura, Kazuto Nakamura, Akira Sato

**Affiliations:** 1 Faculty of Medicine, University of Yamanashi, Shimokato 1110, Chuo, Yamanashi Japan; 2 Department of Cardiology, Faculty of Medicine, University of Yamanashi, Shimokato 1110, Chuo, Yamanashi 409-3898 Japan; 3 Department of Epidemiology and Environmental Medicine, University of Yamanashi, Shimokato 1110, Chuo, Yamanashi Japan

**Keywords:** Improving renal function, Acute heart failure, Heart failure with preserved ejection fraction, Fibrosis-4 index

## Abstract

**Background:**

Improved renal function (IRF) observed in acute heart failure (AHF) is associated with poor prognosis. Since IRF is linked to renal congestion resulting from inadequate decongestion, predicting IRF could enhance management strategies. The Fibrosis-4 (Fib-4) index, originally developed as a marker for liver fibrosis, correlates with hepatic congestion, which is associated with renal congestion, making it a potential predictor of IRF. This study aims to investigate whether the Fib-4 index can predict IRF in patients with AHF and preserved ejection fraction (AHFpEF).

**Methods:**

We analyzed 389 patients hospitalized for AHF between April 2004 and March 2022 at Yamanashi University Hospital. All-cause mortality was monitored for 1 year. IRF was defined as a ≥ 20% increase in the estimated glomerular filtration rate (eGFR) compared to admission levels. Preserved ejection fraction was defined as an ejection fraction ≥ 40%.

**Results:**

IRF was observed in approximately 21% of patients with AHFpEF. Kaplan–Meier analysis showed that patients with IRF had higher mortality rates than those without IRF (p = 0.03, log-rank test). Multivariable analysis for IRF revealed that the eGFR, albumin level, and a Fib-4 index ≥ 3.24 (determined by receiver-operating characteristic curve) on admission were independent predictors of IRF in patients with AHFpEF.

**Conclusion:**

IRF in patients with AHFpEF is associated with poor prognosis. A higher Fib-4 index at admission in AHFpEF can serve as a predictor of IRF.

**Supplementary Information:**

The online version contains supplementary material available at 10.1007/s10157-025-02669-w.

## Introduction

Heart failure (HF) is strongly associated with kidney disease, and renal function affects HF development. Understanding the pathophysiology of cardiorenal syndrome is crucial for the treatment of HF [1]. Renal dysfunction is a common phenomenon in the setting of acute HF (AHF) caused by renal congestion and hypoperfusion [2]; therefore, diuretics should be used with caution in the treatment of AHF complicated by renal dysfunction [3]. Diuretics can influence renal function by both improving it through the removal of renal congestion and worsening it by inducing hypoperfusion via the reduction of preload. The decrease in renal function during AHF is often transient. In such cases, improving renal function (IRF) is observed after admission. IRF is reported to occur in approximately 10–30% of patients with AHF [4, 5]. Furthermore, patients with IRF have been reported to have a high mortality rate [4–6]. IRF is often associated with pre-treatment renal dysfunction in heart failure and is frequently linked to persistent congestion at discharge, both of which are known to be associated with poor prognosis. Almost half of hospitalized patients with AHF have preserved ejection fraction [7].Although previous studies have examined IRF in AHF, no studies have focused on HF with a preserved ejection fraction (HFpEF).

Renal congestion is a more important cause of renal dysfunction in AHF than hypoperfusion [8]. Even if renal function is impaired, adequate treatment of congestion with diuretics may improve the prognosis in these patients; therefore, IRF prediction is important. However, no method has been reported for predicting IRF. Renal congestion can be diagnosed using renal ultrasonography [9]. However, to date, no study has identified a simple marker for renal congestion. The Fibrosis-4 index (Fib-4 index) is a simple marker of liver fibrosis, calculated by age, aspartate transaminase (AST) and alanine aminotransferase (ALT) levels, and platelet count [10].The Fib-4 index is also thought to be related to hepatic congestion and right atrial pressure in AHF [11]. Renal congestion is also caused by increased pressure in the right atrium [9], and is complicated by liver congestion [12]. Therefore, the Fib-4 index may be an indicator of IRF caused by renal congestion.

In this study, we investigated the prognostic value of IRF and whether the Fib-4 index on admission could predict IRF in patients with AHF with preserved ejection fraction (AHFpEF).

## Material and methods

### Study population

**Fig. 1:**
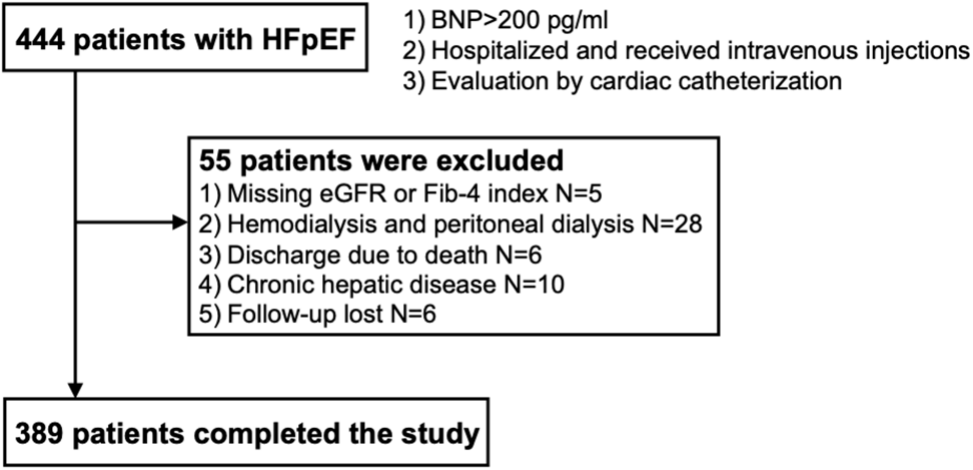
Study design. A total of 389 patients were included in the final analysis. *BNP* B-type natriuretic peptide, *eGFR* estimated glomerular filtration rate, *Fib-4 index* fibrosis-4 index

This study examined 444 patients with AHFpEF and the evaluation was performed using cardiac catheterization who were admitted to the Cardiology Department of the University of Yamanashi Hospital between April 1, 2004, and March 31, 2022. All patients presented with symptoms, signs, and diagnostic findings of HF, had a brain natriuretic peptide (BNP) level of ≥ 200 pg/ml, were treated with intravenous treatments such as inotropes (dobutamine or milrinone: 23.9%), nitrates (isosorbide dinitrate: 88.2%), or diuretics (furosemide or carperitide: 26.7%), and had a left ventricular ejection fraction (LVEF) ≥ 40%. Cardiac catheterization was performed for the assessment of ischemic heart disease and evaluation of cardiac function before hospital discharge. This is a cohort study conducted on patients who underwent cardiac catheterization at the University of Yamanashi Hospital. Patients with severe renal dysfunction or those for whom the attending physician judged, based on their general condition, that cardiac catheterization should not be performed were not included in this study. The exclusion criteria were as follows: (1) missing estimated glomerular filtration rate (eGFR) or Fib-4 index on admission; (2) receiving hemodialysis and peritoneal dialysis daily; (3) discharge due to death; (4) other concomitant causes of hepatic disease, such as viral hepatitis, hepatic cirrhosis, and hepatic tumor; and (5) follow-up lost (Fig. 1). Finally, 389 patients were enrolled based on the inclusion and exclusion criteria. This study was approved by the Ethics Committee of the University of Yamanashi Hospital and conducted in accordance with the 1975 Helsinki Declaration.

### Prospective study

After baseline data were acquired at our hospital, all study patients received medical treatment, including medications and lifestyle advice, according to the Japanese Society of Cardiology (JCS) guidelines. The study participants were followed-up at a hospital or clinic for 12 months, or until all-cause death occurred. The time to the first event was retrospectively evaluated, and all causes of death were confirmed using hospital records. Investigators who were not informed of the baseline clinical characteristics checked for accuracy, consistency, and completeness of follow-up. The endpoint was all-cause death within 1 year of discharge. Three investigators (K.I., Y.W., and Y.I.) checked all data, performed the analysis, and secured the data files.

### Laboratory measurements

Venous blood was obtained from all patients at the time of admission and on the morning of discharge after a 12 h overnight fast. Serum and urine creatinine levels were measured using the creatininase-HMMPS method. Plasma BNP levels were measured using fluorescent enzyme immunoassays. The urinary protein was measured using the pyrogallol red colorimetric method. The LVEF was evaluated using transthoracic echocardiography. The eGFR was calculated as follows: eGFR (ml ⋅ min^−1^⋅ 1.73 m^−2^) = 194 × serum creatinine^−1.094^ × age^−0.287^ (× 0.739 if female). The Fib-4 index was calculated as follows: age (years) × AST (U/L)/ [ALT (U/L)^1/2^ × platelet count (10^3^/µL)].

A preserved ejection fraction was defined as an LVEF ≥ 40%. IRF was defined as a ≥ 20% increase in the eGFR from admission to discharge [5, 6].

### Statistical analysis

Data were expressed as median and interquartile range (IQR) or frequency (%). The Shapiro–Wilk test demonstrated that age, body mass index (BMI), hospitalization days, eGFR, LVEF, blood urea nitrogen (BUN), serum creatinine, serum sodium, serum potassium, serum chloride, BNP, hemoglobin, albumin, AST, and ALT levels, platelet count, cardiac index, aortic pressure, main pulmonary artery pressure, pulmonary capillary wedge pressure, right atrial pressure, and Fib-4 index were not normally distributed, these were therefore expressed as medians and interquartile ranges (IQRs). Median values were compared using the Mann–Whitney U test. Differences in frequencies were evaluated using the Chi-squared test. The association between the change in eGFR from admission to discharge and 1-year mortality, and the associated 95% confidential interval (CIs) were examined using restricted cubic splines with five knots. Kaplan–Meier survival analysis was used to compare between groups with and without IRF.

The cutoff levels of the Fib-4 index as a predictor of IRF were identified using receiver operating characteristic (ROC) curves. Specifically, the cutoff value was determined as the value of the Fib-4 index, which was the Youden's index (sensitivity + specificity – 1) that reached the highest value on the ROC curve. The predictive values of clinical parameters were assessed using univariate and multivariate logistic regression analyses. Univariate logistic regression analysis included age, male sex, BMI, LVEF, smoking status, diabetes mellitus, atrial fibrillation, eGFR, BNP, albumin, and hemoglobin level, and Fib-4 index. Univariate and multivariate logistic regression analyses were used to identify a 1-standard deviation increase in continuous variables. The presence of dichotomous variables was coded as 1, and the absence as 0. All the presented probability values were obtained via two-tailed tests, with statistical significance inferred at p ≤ 0.05. Statistical analysis was performed using EZR, a modified version of the R commander designed to add statistical functions frequently used in biostatistics.

## Result

### Baseline characteristics of patients

In total, 389 patients were enrolled and completed the follow-up study. During the follow-up period, 38 (9.8%) all-cause deaths occurred, of these deaths, 15 (39.5%), 2 (5.3%), 4 (10.5%), and 17 (44.7%) were due to HF, myocardial infarction, cerebrovascular disease, and other causes (including unknown causes of death), respectively. The mean age of the study patients was 77 (IQR 68–82) years, 61.4% were male, 60.9% had hypertension, 29.8% had atrial fibrillation, 34.2% had diabetes mellitus, and the mean hospitalization duration was 17 (12–24) days. Significant valvular heart disease, defined as moderate or greater severity on transthoracic echocardiography, was diagnosed in 131 patients (33.7%). This included severe aortic stenosis (AS) in 13 patients, aortic regurgitation (AR) in 6 patients, and mitral regurgitation (MR) in 12 patients. Among these patients, 15 are scheduled to undergo surgical intervention. The median eGFR was 53.3 (IQR 38.5–70.4) mL/min/1.73 m^2^ on admission, and 52.5 (40.0–67.6) mL/min/1.73 m^2^ at discharge. The median Fib-4 index was 3.15 (IQR 2.12–5.85) on admission, and 1.80 (IQR 1.23–2.58) at discharge.

The baseline characteristics of the study patients with and without all-cause mortality are shown in Table 1. Compared with patients without all-cause death, patients with all-cause death were older and had lower hemoglobin and eGFR on admission and at discharge, higher BUN and Fib-4 levels at discharge, and longer hospitalization days. There was no significant difference in urinary protein levels between patients with and without event.

**Table 1:** Baseline characteristics of study patients (with or without event)

	All patients	Without event	With event	P value
Variables	N = 389	N = 351 (90.2%)	N = 38 (9.8%)	
Age, (years)	77 (68–82)	76 (67–82)	82 (78–86)	< 0.01
Male sex, n (%)	239 (61.4)	212 (60.4)	27 (71.1)	0.22
BMI, kg/m^2^	22.2 (20.3–24.5)	22.3 (20.3–24.5)	21.5 (19.6–24.1)	0.14
Hypertension, n (%)	237 (60.9)	212 (60.4)	25 (65.8)	0.60
Atrial fibrillation, n (%)	116 (29.8)	101 (28.8)	15 (39.5)	0.19
Diabetes mellitus, n (%)	133 (34.2)	116 (33.0)	17 (44.7)	0.15
Smoke, n (%)	155 (39.8)	137 (39.0)	18 (47.4)	0.38
Ischemic heart disease, n (%)	250 (64.3)	227 (65.0)	23 (62.2)	0.72
Valvular heart disease, n (%)	131 (33.7)	116 (33.0)	15 (39.5)	0.47
IRF, n (%)	82 (21.1)	69 (19.7)	13 (34.2)	0.06
Hospitalization days	17 (12–24)	17 (12–23)	21 (14–38)	0.02
BUN, (mg/dL)	20.9 (14.9–27.8)	19.8 (14.7–26.3)	26.0 (22.5–38.1)	< 0.01
Serum creatinine on admission, (mg/dL)	0.97 (0.75–1.31)	0.94 (0.73–1.25)	1.10 (0.93–1.55)	< 0.01
Serum creatinine at discharge, (mg/dL)	0.97 (0.77–1.23)	0.96 (0.74–1.23)	1.10 (0.96–1.34)	0.02
eGFR on admission, (mL/min/1.73 m^2^)	53.3 (38.5–70.4)	54.2 (39.9–72.2)	44.6 (30.6–55.8)	< 0.01
eGFR at discharge, (mL/min/1.73 m^2^)	52.5 (40.0–67.6)	53.5 (40.8–68.2)	45.4 (35.7–55.7)	0.03
BNP, (pg/mL)	458 (295–808)	458 (296–808)	475 (290–800)	0.96
LVEF, (%)	53 (46–63)	53 (46–63)	51 (45–61)	0.26
Hemoglobin, (g/dL)	12.7 (11.1–14.1)	12.8 (11.3–14.2)	12.0 (10.7–13.0)	0.03
Albumin, (g/dL)	3.7 (3.4–4.0)	3.7 (3.4–4.0)	3.6 (3.3–3.9)	0.24
Serum sodium, (mg/dL)	139 (137–141)	139 (137–141)	140 (138–142)	0.28
Serum potassium, (mg/dL)	4.1 (3.8–4.5)	4.1 (3.8–4.5)	4.3 (3.9–4.7)	0.12
Serum chloride, (mg/dL)	105 (102–107)	105 (102–107)	106 (103–107)	0.10
AST (U/L) on admission	37 (24–89)	38 (24–89)	33 (23–64)	0.43
AST (U/L) at discharge	24 (19–32)	24 (19–32)	27 (20–35)	0.20
ALT (U/L) on admission	25 (16–41)	26 (16–42)	23 (19–36)	0.46
ALT (U/L) at discharge	21 (13–34)	21 (13–34)	20 (10–35)	0.47
Platelet count (× 10^3^/μL) on admission	195 (150–239)	196 (152–241)	170 (131–225)	0.09
Platelet count (× 10^3^/μL) at discharge	226 (174–297)	226 (175–300)	227 (156–283)	0.34
Urine total protein, (mg/dL)	6 (5–15)	6 (5–14)	7 (5–23)	0.22
Urine creatinine, (mg/dL)	55.3 (42.4–80.1)	55.3 (42.4–82.1)	57.2 (42.5–73.2)	0.73
Urine protein-to-creatinine Ratio (g/g Cr)	0.13 (0.09–0.25)	0.13 (0.09–0.25)	0.13 (0.12–0.23)	0.47
Cardiac index	2.7 (2.3–3.2)	2.7 (2.3–3.2)	2.5 (2.3–2.9)	0.32
Ao, mean	94 (83–110)	95 (83–110)	94 (82–109)	0.52
mPA, mean	23 (17–29)	23 (17–29)	23 (16–31)	0.77
PCW, mean	17 (11–23)	16 (11–23)	19 (11–24)	0.68
RA, mean	7.0 (4.0–11.0)	7.0 (5.0–11.0)	7.0 (3.5–11.5)	0.70
Fib-4 index on admission	3.15 (2.12–5.85)	3.14 (2.12–5.73)	3.65 (2.09–7.04)	0.36
Fib-4 index at discharge	1.80 (1.23–2.58)	1.76 (1.22–2.56)	2.39 (1.58–3.91)	< 0.01
Medication, n (%)				
ACEI/ARB, (%)	197 (50.6)	178 (50.7)	19 (50.0)	1
β-Blocker, (%)	161 (41.4)	141 (40.2)	20 (52.6)	0.17
Loop diuretic, (%)	188 (48.3)	168 (47.9)	20 (52.6)	0.61
MRA, (%)	83 (21.3)	75 (21.4)	8 (21.1)	1

Data were expressed as median and interquartile range (IQR) or frequency (%); P-values represent comparisons between patients with and without events

*ACEI* angiotensin-converting enzyme inhibitor, *ALT* alanine aminotransferase, *Ao* aorta, *ARB* angiotensin II receptor blocker, *AST* aspartate aminotransferase, *BMI* body mass index, *BNP* B-type natriuretic peptide, *BUN* blood urea nitrogen, *eGFR* estimated glomerular filtration rate, *Fib-4 index* fibrosis-4 index, *LVEF*, left ventricular ejection fraction, *mPA* mean pulmonary artery pressure, *MRA* mineralocorticoid receptor antagonist, *PCW* pulmonary capillary wedge pressure, *RA* right atrial pressure

### Clinical characteristics of patients with and without IRF

IRF occurred in 82 (21.1%) patients. The clinical characteristics of patients with and without IRF are summarized in Table 2. Patients with IRF were older and had longer hospitalization days, higher BUN and BNP levels, and lower hemoglobin and albumin levels. Moreover, patients with IRF had a lower eGFR on admission, and higher Fib-4 index on admission and at discharge (Table 2). There was no significant difference in eGFR at discharge and  urinary protein levels between patients with and without IRF. The weight reduction during hospitalization was significantly greater in the patients with IRF compared to without IRF (Supplementary Fig. 1).

**Table 2:** Baseline characteristics of study patients (with or without IRF)

	All patients	Non-IRF	IRF	P-value
Variables	N = 389	N = 307 (78.9%)	N = 82 (21.1%)	
Mortalities (1 year), n (%)	38 (9.8)	25 (8.1)	13 (15.9)	0.06
Age, (years)	77 (68–82)	76 (67–82)	78 (70–84)	0.02
Male sex, n (%)	239 (61.4)	193 (62.9)	46 (56.1)	0.31
BMI, kg/m^2^	22.2 (20.3–24.5)	22.4 (20.4–24.7)	21.6 (19.7–24.0)	0.05
Hypertension, n (%)	237 (60.9)	186 (60.6)	51 (62.2)	0.90
Atrial Fibrillation, n (%)	116 (29.8)	86 (28.0)	30 (36.6)	0.14
Diabetes mellitus, n (%)	133 (34.2)	107 (34.9)	26 (31.7)	0.69
Smoke, n (%)	155 (39.8)	126 (41.0)	29 (35.4)	0.38
Ischemic heart disease, n (%)	250 (64.3)	201 (65.9)	49 (60.5)	0.36
Valvular heart disease, n (%)	131 (33.7)	97 (31.6)	34 (41.5)	0.11
Hospitalization days	17 (12–24)	16 (12–23)	20 (15–32)	< 0.01
BUN, (mg/dL)	20.9 (14.9–27.8)	18.8 (14.3–24.2)	34.4 (24.8–43.8)	< 0.01
Serum creatinine on admission, (mg/dL)	0.97 (0.75–1.31)	0.90 (0.70–1.12)	1.39 (1.03–1.83)	< 0.01
Serum creatinine at discharge, (mg/dL)	0.97 (0.77–1.23)	0.97 (0.79–1.23)	0.96 (0.72–1.23)	0.35
eGFR on admission, (mL/min/1.73 m^2^)	53.3 (38.5–70.4)	56.8 (44.7–74.1)	36.8 (22.2–49.1)	< 0.01
eGFR at discharge, (mL/min/1.73 m^2^)	52.5 (40.0–67.6)	52.5 (40.8–66.7)	53.2 (39.1–68.9)	0.74
BNP, (pg/mL)	458 (295–808)	425 (282–744)	624 (389–1139)	< 0.01
LVEF, (%)	53 (46–63)	53 (45–62)	56 (47–68)	0.08
Hemoglobin, (g/dL)	12.7 (11.1–14.1)	12.9 (11.5–14.4)	11.4 (10.1–13.0)	< 0.01
Albumin, (g/dL)	3.7 (3.4–4.0)	3.7 (3.4–4.1)	3,4(3.1–3.8)	< 0.01
Serum sodium, (mg/dL)	139 (137–141)	139 (137–141)	139 (135–141)	0.03
Serum potassium, (mg/dL)	4.1 (3.8–4.5)	4.1 (3.8–4.4)	4.4 (3.9–4.8)	< 0.01
Serum chloride, (mg/dL)	105 (102–107)	105 (102–107)	104 (101–107)	0.09
AST (U/L) on admission	37 (24–89)	36 (24–73)	59 (25–142)	< 0.01
AST (U/L) at discharge	24 (19–32)	24 (19–33)	25 (19–31)	0.90
ALT (U/L) on admission	25 (16–41)	24 (16–38)	31 (17–65)	0.02
ALT (U/L) at discharge	21 (13–34)	22 (13–34)	19 (13–34)	0.53
Platelet count (× 10^3^/μL) on admission	195 (150–239)	198 (156–246)	178 (133–228)	< 0.01
Platelet count (× 10^3^/μL) at discharge	226 (174–297)	231 (180–305)	207 (151–263)	< 0.01
Urine total protein, (mg/dL)	6 (5–15)	5 (5–13)	7 (5–19)	0.11
Urine creatinine, (mg/dL)	55.3 (42.4–80.1)	54.9 (42.3–80.5)	56.6 (43.1–77.3)	0.89
Urine protein-to-creatinine Ratio (g/g Cr)	0.13 (0.09–0.25)	0.13 (0.09–0.23)	0.16 (0.09–0.30)	0.41
Cardiac index	2.7 (2.3–3.2)	2.6 (2.3–3.1)	2.9 (2.4–3.4)	0.05
Ao, mean	94 (83–110)	96 (85–111)	86 (71–97)	< 0.01
mPA, mean	23 (17–29)	23 (17–29)	24 (17–31)	0.51
PCW, mean	17 (11–23)	17 (11–22)	16 (11–27)	0.99
RA, mean	7.0 (4.0–11.0)	7 (4–10)	9 (5–13)	0.05
Fib-4 index on admission	3.15 (2.12–5.85)	2.95 (1.98–5.22)	4.52 (2.70–7.98)	< 0.01
Fib-4 index at discharge	1.80 (1.23–2.58)	1.70 (1.20–2.53)	2.19 (1.43–3.59)	< 0.01
Medication, n (%)				
ACEI/ARB, (%)	197 (50.6)	161 (52.4)	36 (43.9)	0.17
β-Blocker, (%)	161 (41.4)	134 (43.6)	27 (32.9)	0.10
Loop diuretic, (%)	188 (48.3)	147 (47.9)	41 (50.0)	0.80
MRA, (%)	83 (21.3)	70 (22.8)	13 (15.9)	0.22

P values represent comparisons between patients with and without IRF

*ACEI* angiotensin-converting enzyme inhibitor, *ALT* alanine aminotransferase, *Ao* aorta, *ARB* angiotensin II receptor blocker, *AST* aspartate aminotransferase, *BMI* body mass index, *BNP* B-type natriuretic peptide, *BUN* blood urea nitrogen, *eGFR* estimated glomerular filtration rate, *Fib-4 index* fibrosis-4 index, *LVEF*, left ventricular ejection fraction, *mPA* mean pulmonary artery pressure, *MRA* mineralocorticoid receptor antagonist, *PCW* pulmonary capillary wedge pressure, *RA* right atrial pressure

The analysis of patients with preadmission data and changes in eGFR from preadmission to discharge is shown in Supplementary Fig. 2. Patients with IRF exhibited worsening renal function from preadmission to admission. By contrast, in an analysis of a limited number of patients with preadmission data, there was no difference in the eGFR before admission between patients with or without IRF.

### Survival analysis

**Fig. 2:**
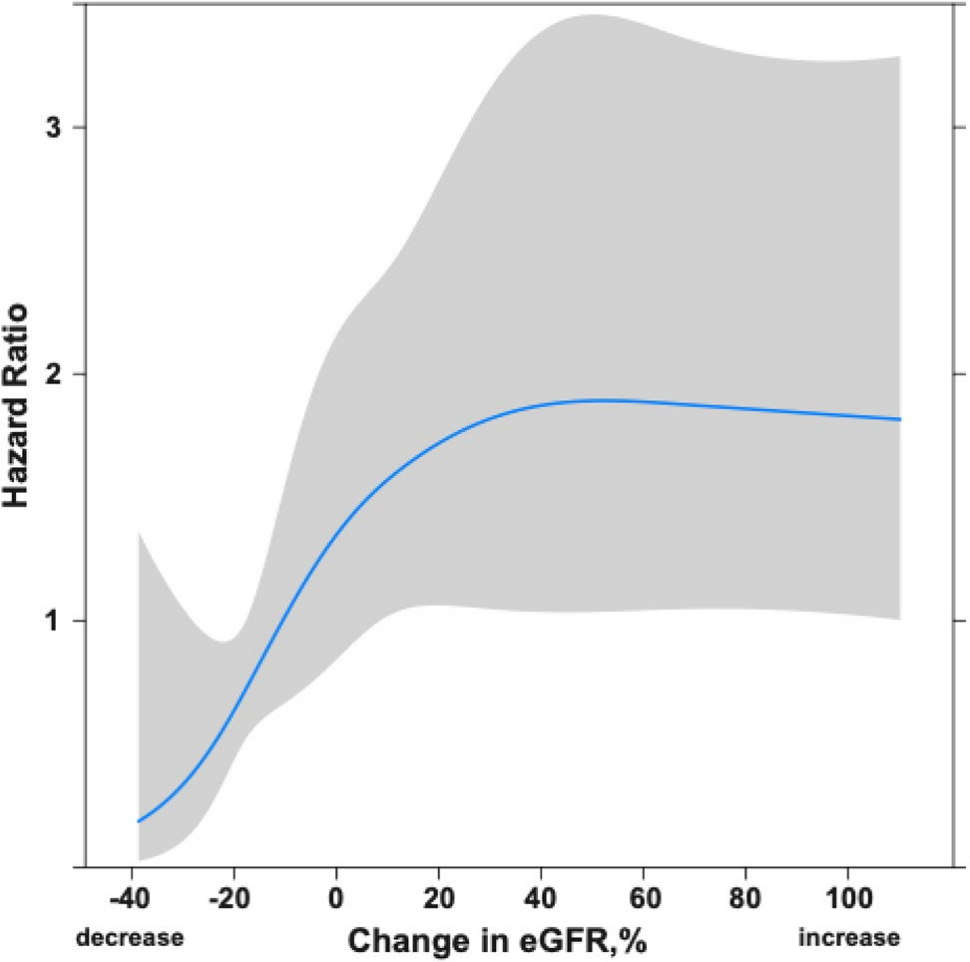
Restricted cubic spline analysis for 1-year mortality by change in eGFR from admission to discharge. Hazard ratios are indicated by solid lines and 95% confidence intervals by shaded areas. *eGFR* estimated glomerular filtration rate

**Fig. 3:**
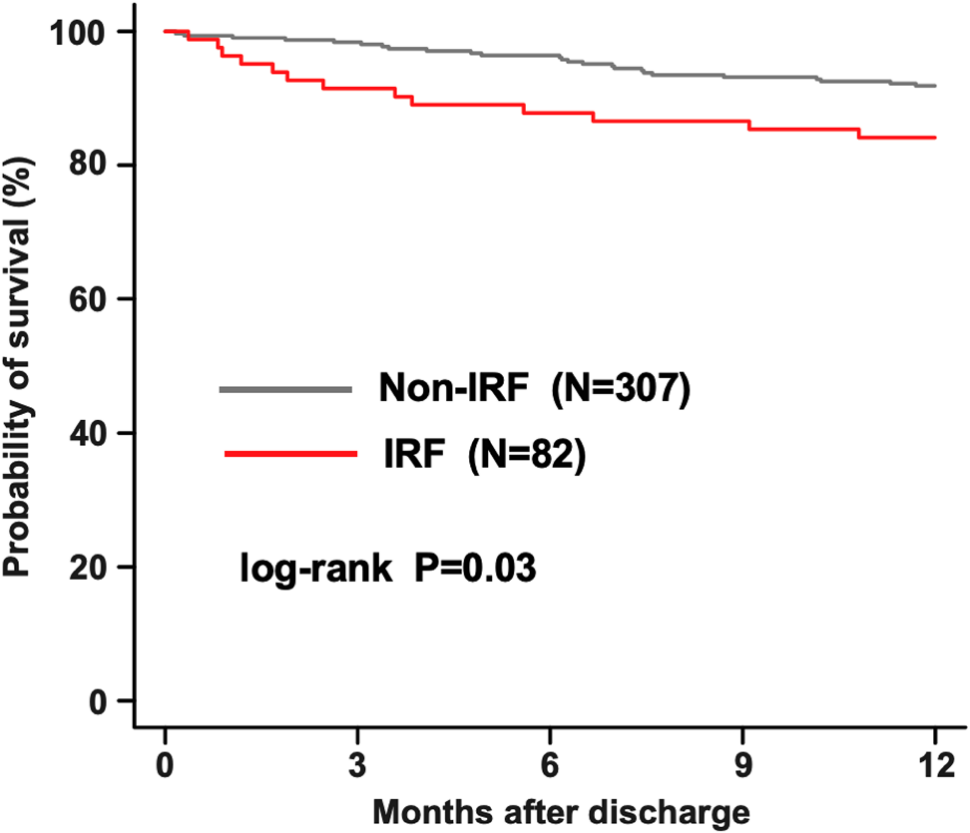
Kaplan–Meier analysis for the probability of all cause death according to the presence or absence of IRF. *IRF* improving renal function

A restricted cubic spline analysis for 1-year mortality based on changes in eGFR from admission to discharge is shown in Fig. 2. Decreased eGFR during hospitalization was associated with a lower risk of mortality, and an increased change in eGFR—up to 40%—was associated with the 1-year mortality risk. When the clinical outcomes were stratified according to the presence or absence of IRF, Kaplan–Meier analysis showed that patients with IRF had a significantly higher 1-year mortality than those without IRF (15.9% vs. 8.1%, P = 0.03; Fig. 3).

### Predictive value of Fib-4 index for IRF

**Fig. 4:**
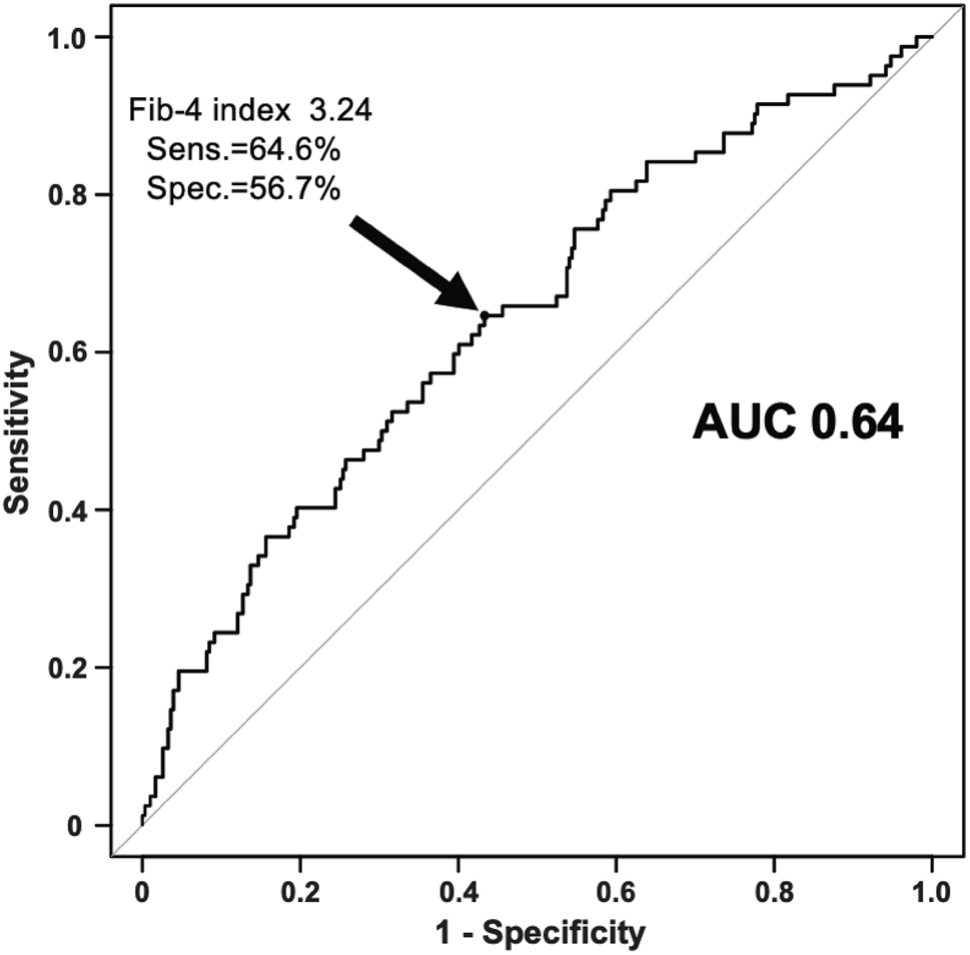
Receiver-operating characteristic (ROC) curve of levels of Fib-4 index for IRF The ROC curve was used to obtain the optimal cutoff level of the Fib-4 index for the prediction of IRF. *AUC* area under the curve, *Fib-4 index* fibrosis-4 index, *IRF* improving renal function

Based on the ROC curve analysis, the cutoff value of the Fib-4 index on admission for predicting IRF was 3.24 with a sensitivity of 64.6% and specificity of 56.7% for predicting future IRF (Fig. 4). The area under the curve was 0.64 (Fig. 4). Clinical characteristics of patients with a Fib-4 index < 3.24 and ≥ 3.24 on admission are summarized in Supplementary Table 1. Patients with a higher Fib-4 index on admission were older and had higher BUN levels and Fib-4 index at discharge.

Univariate logistic regression analysis showed that age (odds ratio [OR]: 1.35, 95% CI 1.03–1.76), albumin level (OR: 0.59; 95% CI 0.46–0.75), eGFR (OR: 0.27; 95% CI 0.19–0.39), BNP level (OR: 1.63; 95% CI 1.28–2.07), hemoglobin level (OR: 0.57; 95% CI 0.44–0.73), and Fib-4 index ≥ 3.24 on admission (OR: 2.36; 95% CI 1.42–3.91) were significant predictors of IRF (Table 3). The predictive values of albumin level, eGFR, and Fib-4 index on admission remained significant in the multivariate logistic regression analysis (Table 3). Moreover, category-free net reclassification improvement (NRI) and integrated discrimination improvement (IDI) demonstrated the additive value of a Fib-4 index ≥ 3.24 on admission to the predictive factor, following albumin level and eGFR on admission with significantly increased probability (NRI: 0.42, P < 0.01; IDI: 0.04, P < 0.01; Table 4).

**Table 3:** Univariate and multivariate logistic regression analysis for IRF in heart failure with preserved ejection fraction

Variables	Univariate analysis			Multivariate analysis		
	OR	95% CI	P value	OR	95% CI	P value
Age, per SD	1.35	1.03–1.76	0.03	0.97	0.72–1.32	0.86
Male sex	0.76	0.46–1.24	0.26			
BMI, per SD	0.79	0.61–1.03	0.09			
LVEF, per SD	1.26	0.99–1.60	0.06			
Smoke	0.79	0.47–1.30	0.35			
DM	0.87	0.52–1.46	0.59			
AF	1.48	0.89–2.48	0.13			
Albumin on admission, per SD	0.59	0.46–0.75	< 0.01	0.68	0.49–0.96	0.03
eGFR on admission, per SD	0.27	0.19–0.39	< 0.01	0.29	0.19–0.43	< 0.01
BNP on admission, per SD	1.63	1.28–2.07	< 0.01	1.21	0.92–1.59	0.18
Hemoglobin on admission, per SD	0.57	0.44–0.73	< 0.01	1.12	0.79–1.58	0.54
Fib-4 index on admission ≥ 3.24	2.36	1.42–3.91	< 0.01	2.66	1.46–4.86	< 0.01

The odds ratios (ORs) and 95% confidence intervals (CIs) for continuous variables were estimated using a 1-standard deviation (SD) increase (per 1-SD)

*ACEI* angiotensin-converting enzyme inhibitor, *ALT* alanine aminotransferase, *Ao* aorta, *ARB* angiotensin II receptor blocker, *AST* aspartate aminotransferase, *BMI* body mass index, *BNP* B-type natriuretic peptide, *BUN* blood urea nitrogen, *eGFR* estimated glomerular filtration rate, *Fib-4 index* fibrosis-4 index, *LVEF*, left ventricular ejection fraction, *mPA* mean pulmonary artery pressure, *MRA* mineralocorticoid receptor antagonist, *PCW* pulmonary capillary wedge pressure, *RA* right atrial pressure

Abbreviations are the same as those in Table 1

**Table 4:** NRI and IDI for the incremental predictive values of the combination of risk factors

	Category-free NRI		IDI	
	Index	P value	Index	P value
eGFR + Albumin	⎼	⎼	⎼	⎼
eGFR + Albumin + Fib-4 index ≥ 3.24	0.42	< 0.01	0.04	< 0.01

*IDI* integrated discrimination improvement, *NRI* net reclassification improvement, *ACEI* angiotensin-converting enzyme inhibitor, *ALT* alanine aminotransferase, *Ao* aorta, *ARB* angiotensin II receptor blocker, *AST* aspartate aminotransferase, *BMI* body mass index, *BNP* B-type natriuretic peptide, *BUN* blood urea nitrogen, *eGFR* estimated glomerular filtration rate, *Fib-4 index* fibrosis-4 index, *LVEF*, left ventricular ejection fraction, *mPA* mean pulmonary artery pressure, *MRA* mineralocorticoid receptor antagonist, *PCW* pulmonary capillary wedge pressure, *RA* right atrial pressure

## Discussion

The major findings of this study were that patients with AHFpEF with IRF have higher mortality, and that the eGFR, albumin level, and a Fib-4 index ≥ 3.24 on admission are independent predictive factors of IRF. Additionally, the Fib-4 index significantly improved the predictive ability for IRF when adding the Fib-4 index to the models of eGFR and albumin. Therefore, the Fib-4 index can predict improved renal function associated with poor prognosis in patients with AHFpEF. According to previous research, patients with AHF with IRF have a higher mortality [4–6]. In the current study, the mortality of patients with IRF at 1-year post discharge was significantly higher than that of patients without IRF when the analysis was limited to AHFpEF. Previous studies have reported that elevated Fib-4 index at both admission and discharge are poor prognostic factors [13–15]. However, in the present study, while the Fib-4 index at discharge was higher in the event group, no significant difference was observed in the Fib-4 index at admission.

Patients with IRF had admission features consistent with a more severe HF presentation, including longer hospitalization days and higher BNP. Additionally, the weight reduction due to treatment was significantly greater, suggesting a higher degree of fluid retention at admission. The right atrial pressure (measured using a right heart catheter) at discharge was higher in patients with IRF than without IRF. Previous studies have shown that elevated right atrial pressure, which is associated with organ congestion and right ventricular failure [16], has been linked to a poorer prognosis [17]. Higher right atrial pressure at discharge in patients with IRF may be a contributing factor to the poor prognosis observed in this study.

In the current study, we revealed that three factors were predictive of IRF: eGFR, albumin level, and Fib-4 index on admission. Furthermore, the addition of the Fib-4 index to the model of eGFR and albumin level increased the predictive ability of IRF. Low eGFR on admission was associated with IRF due to worsening renal function before admission. Hypoalbuminemia, which deteriorates interstitial edema [18] may contribute to renal congestion. In HF, increased right atrial pressure causes edema and organ congestion in the liver and kidneys. The Fib-4 index, which has been developed as a hepatic fibrosis marker, is thought to be associated with hepatic congestion in HF [11]. In the current study, we demonstrated that the Fib-4 index decreased with AHF treatment. This result supports the idea that the Fib-4 index is an indicator of transient hepatic congestion, rather than liver fibrosis, in patients with AHF. While renal congestion is not a simple indicator, like hepatic congestion, it is caused by an increase in right atrial pressure in AHF. Thus, the Fib-4 index — associated with hepatic congestion — may predict IRF due to renal congestion.

The main causes of renal dysfunction in AHF are renal congestion and low-output syndrome [19]. Patients with AHF and impaired renal function on admission who later develop IRF do not achieve adequate decongestion and have a poor prognosis [6]. In the present study, compared with patients without IRF, patients with IRF in this study exhibited significantly higher right atrial pressures and Fib-4 index indices at discharge, suggesting residual congestion. We identified that the Fib-4 index at admission serves as a predictor of IRF in patients with AHFpEF and it may predict IRF due to renal congestion.

Our study has several limitations. First, this was a single-center study; the ratio of the types or severity of HF may be specific to our hospital and may have influenced the results. Second, the relatively small number of enrolled patients limited the statistical power of the study. Third, this did not include patients with advanced CKD (like patients with hemodialysis daily), and therefore its assessment of severe CKD is insufficient. Fourth, patients with AHF, cardiac catheterization was performed for the evaluation of ischemic heart disease and assessment of cardiac function. However, patients with significantly impaired renal function or poor general condition were excluded from this study, because the physician determined that the patient was unsuitable for cardiac catheterization.

Our study demonstrates that IRF is associated with higher mortality, and a Fib-4 index ≥ 3.24 predicts IRF in patients with AHFpEF.

## Supplementary Information

Below is the link to the electronic supplementary material.

Supplementary file1 (DOCX 36 KB)

Supplementary file2 (DOCX 73 KB)

Supplementary file3 (DOCX 20 KB)
