# Supplementary material for: Fibrosis-4 index can predict improved renal function in acute heart failure with preserved ejection fraction
Source: Clin Exp Nephrol. 2025 Apr 7;29(9):1163–71. doi: 10.1007/s10157-025-02669-w (PMC12441086; doi:10.1007/s10157-025-02669-w)
Supplement: Supplementary file 1 — Supplementary file1 (DOCX 36 KB) [file 10157_2025_2669_MOESM1_ESM.docx]

**Supplementary Figure 1**.

Weight reduction during hospitalization in patients with and without IRF

The percentage decrease in body weight at discharge compared to admission was reported.

*P < 0.01

IRF, improving renal function
