# Supplementary material for: Fibrosis-4 index can predict improved renal function in acute heart failure with preserved ejection fraction
Source: Clin Exp Nephrol. 2025 Apr 7;29(9):1163–71. doi: 10.1007/s10157-025-02669-w (PMC12441086; doi:10.1007/s10157-025-02669-w)
Supplement: Supplementary file 2 — Supplementary file2 (DOCX 73 KB) [file 10157_2025_2669_MOESM2_ESM.docx]

**Supplementary Figure 2**. Comparison of eGFR in patients with or without IRF before admission, on admission, and at discharge.


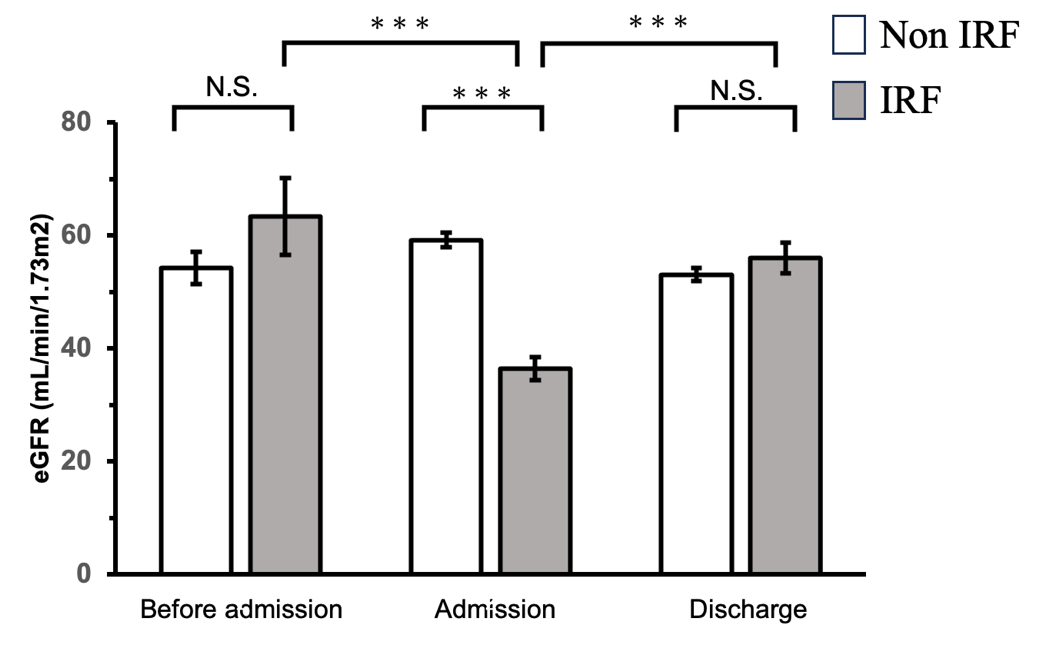


Only patients with preadmission data were analyzed.

Patients without IRF (non-IRF): N=60, patients with IRF: N=8

***P < 0.01

eGFR, estimated glomerular filtration rate; IRF, improving renal function
