# Supplementary material for: Fibrosis-4 index can predict improved renal function in acute heart failure with preserved ejection fraction
Source: Clin Exp Nephrol. 2025 Apr 7;29(9):1163–71. doi: 10.1007/s10157-025-02669-w (PMC12441086; doi:10.1007/s10157-025-02669-w)
Supplement: Supplementary file 3 — Supplementary file3 (DOCX 20 KB) [file 10157_2025_2669_MOESM3_ESM.docx]

**Supplementary Table 1.** Baseline characteristics of study patients with and without a Fib-4 index ≥3.24

|  | All patients | Fib-4 index <3.24 | Fib-4 index ≥3.24 | P-value |
| --- | --- | --- | --- | --- |
| variables | N=389 | N=202 (51.9%) | N=187(48.1%) |  |
| Mortalities (1 year), n (%) | 38 (9.8) | 18 (8.9) | 20 (10.7) | 0.61 |
| Age, (years) | 77 (68-82) | 75 (65-81) | 78 (70-83) | <0.01 |
| Male sex, n (%) | 239 (61.4) | 131 (64.9) | 108 (57.8) | 0.18 |
| BMI, kg/m^2^ | 22.2 (20.3-24.5) | 22.4 (20.7-25.1) | 22.1 (19.9-24.0) | 0.04 |
| Hypertension, n (%) | 237 (60.9) | 129 (63.9) | 108 (57.8) | 0.25 |
| Atrial Fibrillation, n (%) | 116 (29.8) | 62 (30.7) | 54 (28.9) | 0.74 |
| Diabetes mellitus, n (%) | 133 (34.2) | 79 (39.1) | 54 (28.9) | 0.04 |
| Smoke, n (%) | 155 (39.8) | 88 (43.6) | 67 (35.8) | 0.12 |
| Ischemic heart disease, n (%) | 250 (64.3) | 115 (57.5) | 135 (72.6) | <0.01 |
| Valvular heart disease, n (%) | 131 (33.7) | 70 (34.7) | 61 (32.6) | 0.75 |
| Improving renal function, n (%) | 82 ( 21.1) | 29 (14.4) | 53 (28.3) | <0.01 |
| Hospitalization days | 17 (12-24) | 17 (12-24) | 18 (13-24) | 0.23 |
| BUN, (mg/dL) | 20.9 (14.9-27.8) | 19.2 (14.8-25.8) | 22.5 (15.4-31.7) | 0.02 |
| Serum creatinine on admission, (mg/dL) | 0.97 (0.75-1.31) | 0.93 (0.75-1.27) | 1.03 (0.75-1.33) | 0.59 |
| Serum creatinine at discharge, (mg/dL) | 0.97 (0.77-1.23) | 0.98 (0.80-1.26) | 0.97 (0.73-1.23) | 0.15 |
| eGFR on admission, (mL/min/1.73 m^2^) | 53.3 (38.5-70.4) | 55.7 (38.5-72.6) | 51.4 (38.7-65.8) | 0.20 |
| eGFR at discharge, (mL/min/1.73 m^2^) | 52.5 (40.0-67.6) | 51.7 (39.1-66.4) | 54.0 (40.8-68.3) | 0.45 |
| BNP, (pg/mL) | 458 (295-808) | 468 (290-831) | 454 (298-795) | 0.83 |
| LVEF, (%) | 53 (46-63) | 52 (45-63) | 55 (47-63) | 0.13 |
| Hemoglobin, (g/dL) | 12.7 (11.1-14.1) | 12.6 (11.1-14.3) | 12.8 (11.2-14.0) | 0.41 |
| Albumin, (g/dL) | 3.7 (3.4-4.0) | 3.7 (3.3-4.0) | 3.7 (3.4-4.0) | 0.62 |
| Serum sodium, (mg/dL) | 139 (137-141) | 140 (138-141) | 139 (136-141) | <0.01 |
| Serum potassium, (mg/dL) | 4.1 (3.8-4.5) | 4.1 (3.8-4.5) | 4.2 (3.8-4.5) | 0.99 |
| Serum chloride, (mg/dL) | 105 (102-107) | 106 (103-107) | 105 (102-107) | 0.02 |
| AST (U/L) on admission | 37 (24-89) | 25 (20-36) | 89 (44-148) | <0.01 |
| AST (U/L) at discharge | 24 (19-32) | 23 (18-31) | 25 (21-33) | 0.02 |
| ALT (U/L) on admission | 25 (16-41) | 19 (13-32) | 34 (21-60) | <0.01 |
| ALT (U/L) at discharge | 21 (13-34) | 21 (12-35) | 20 (15-34) | 0.88 |
| Platelet count (×10^3^/μL) on admission | 195 (150-239) | 224 (175-256) | 165 (134-203) | <0.01 |
| Platelet count (×10^3^/μL) at discharge | 226 (174-297) | 233 (186-310) | 216 (167-281) | 0.02 |
| Cardiac index | 2.7 (2.3-3.2) | 2.7 (2.3-3.3) | 2.6 (2.3-3.0) | 0.37 |
| Ao, mean | 94 (83-110) | 95 (84-110) | 94 (83-109) | 0.63 |
| mPA, mean | 23 (17-29) | 23 (17-30) | 23 (17-29) | 0.69 |
| PCW, mean | 17 (11-23) | 17 (11-23) | 17 (11-23) | 0.87 |
| RA, mean | 7.0 (4.0-11.0) | 7 (4-11) | 8 (5-11) | 0.29 |
| Fib-4 index on admission | 3.15 (2.12-5.85) | 2.16 (1.58-2.64) | 6.20 (4.39-9.00) | <0.01 |
| Fib-4 index at discharge | 1.80 (1.23-2.58) | 1.68 (1.10-2.36) | 1.93 (1.38-3.09) | <0.01 |
| Medication, n (%) |  |  |  |  |
| ACEI/ARB, (%) | 197 (50.6) | 108 (53.5) | 89 (47.6) | 0.27 |
| β-Blocker, (%) | 161 (41.4) | 87 (43.1) | 74 (39.6) | 0.54 |
| Loop diuretic, (%) | 188 (48.3) | 106 (52.5) | 82 (43.9) | 0.10 |
| MRA, (%) | 83 (21.3) | 50 (24.8) | 33 (17.6) | 0.11 |

P-values, comparison between patients without and with a Fib-4 index ≥3.24 on admission.

ACEI, angiotensin-converting enzyme inhibitor; ALT, alanine aminotransferase; Ao, aorta; ARB, angiotensin II receptor blocker; AST, aspartate aminotransferase; BMI, body mass index; BNP, B-type natriuretic peptide; BUN, blood urea nitrogen; eGFR, estimated glomerular filtration rate; Fib-4 index, fibrosis-4 index; LVEF; left ventricular ejection fraction; mPA, mean pulmonary artery pressure; MRA, mineralocorticoid receptor antagonist PCW, pulmonary capillary wedge pressure RA, right atrial pressure
